# M2 Macrophages derived exosomes promoted Periodontal ligament stem cells osteogenic differentiation through secreting CCL18

**DOI:** 10.1016/j.clinsp.2026.100916

**Published:** 2026-03-27

**Authors:** Zhongqiang Feng, Yanyan Zhang, Jun Zhang, Zhangyan Ye

**Affiliations:** Department of Stomatology, The People's Hospital of Pingyang, Pingyang County, Wenzhou City, Zhejiang Province, China

**Keywords:** Chemokine CCL18, Stem cells, Osteogenesis, Exosomes, Macrophages

## Abstract

**Purpose:**

This research aimed to examine the impact of M2 Macrophages-derived exosomes (M2-exo) on the osteogenic differentiation of Periodontal Ligament Stem Cells (PDLSCs).

**Methods:**

Human monocytes THP-1 were used to induce the M1 and M2 polarization. The PDLSCs were co-cultured with M1 and M2 type THP-1, and subjected to M2-exo treatment, respectively. The osteogenic differentiation of PDLSCs was analyzed through ALP and ARS staining. The mRNA expression of biomarkers of M1 and M2 macrophages, OPN, Runx2, ALP and CCL18 was evaluated by RT-qPCR assay.

**Results:**

The findings suggested that M1 type THP-1 inhibited the steogenic differentiation of PDLSCs and M2 type THP-1 promoted it. M2-exo also promoted steogenic differentiation of PDLSCs and increased the OPN, Runx2 and ALP mRNA levels. Furthermore, CCL18 levels were increased in M2 type THP-1 and M2-exo treated PDLSCs. After CCL18 knockdown, the role of M2-exo in PDLSCs was reversed.

**Conclusion:**

In conclusion, this study confirmed that M2-exo derived exosomes facilitated PDLSCs' osteogenic differentiation through secreting CCL18.

## Highlights

• M2-exo facilitated osteogenic differentiation of hPDLSCs.
• CCL18 was upregulated in M2-exo and M2-exo treated hPDLSCs.
• CCL18 knockdown reversed the impact of M2-exo in hPDLSCs.

## Introduction

Periodontal Disease (PD), a chronic inflammatory disorder, is defined by the degradation of the tissues supporting the teeth, including the gingiva, periodontal ligament, and alveolar bone.^1^ Current clinical treatments for periodontitis ‒ such as scaling and root planing, antibiotic therapy, and surgical intervention ‒ primarily aim to control infection and halt disease progression.^2^ However, these approaches often fall short in achieving predictable and complete regeneration of the lost periodontal tissues, particularly the functional restoration of the periodontal ligament and alveolar bone.^3^ This limitation underscores the urgent need for novel therapeutic strategies that can effectively promote periodontal tissue repair and regeneration.

The pathogenesis of PD is characterized by intricate interplays between the host's immune system and the oral microbiota.^4^ Within the diverse cellular constituents of the innate immune response, macrophages are crucial for both the onset and progression of PD.^5^ Macrophages are highly plastic cells that can adapt to their microenvironment through a process known as polarization. Traditionally, macrophages have been classified into two main functional states: M1 (classically activated) and M2 (alternatively activated).^6^ M1 macrophages are associated with pro-inflammatory responses and tissue destruction, whereas M2 macrophages exhibit anti-inflammatory properties and contribute to tissue repair and regeneration.^7^ Recent studies have highlighted the importance of M2 macrophages in the resolution of inflammation and tissue regeneration in PD.^8^

Exosomes, small Extracellular Vesicles (EVs) released by various cell types, including macrophages, contain bioactive molecules such as proteins, lipids, and nucleic acids, and play a pivotal role in intercellular communication.^9^, ^10^, ^11^ Notably, exosomes derived from M2 macrophages (M2-exos) have emerged as promising mediators of tissue repair and immunomodulation in various regenerative contexts.^12^ For instance, M2-exos have been shown to enhance osteogenesis in bone defect models,^13^ promote diabetic fracture healing,^11^ and facilitate neural and cutaneous wound repair.^14^ In the dental field, M2-exos have been reported to enhance the regeneration of the dentin-pulp complex and suppress inflammation in dental pulp cells.^15^ These findings collectively underscore the therapeutic potential of M2-exos in regenerative medicine.

Periodontal Ligament Stem Cells (PDLSCs), a type of mesenchymal stem cell, exhibit multipotent differentiation potential, including the ability to differentiate into osteoblasts, which is critical for periodontal bone regeneration.^16^^,^^17^^,^^18^ However, the inflammatory microenvironment in periodontitis often impairs the osteogenic capacity of PDLSCs. While the immunomodulatory and regenerative functions of M2-exos have been increasingly recognized, the specific mechanisms by which they influence the osteogenic differentiation of PDLSCs remain largely unexplored.

CCL18 is a chemokine primarily produced by alternatively activated M2 macrophages and has been implicated in several physiological and pathological processes, including inflammation, angiogenesis, and tissue remodeling.^19^^,^^20^ In the context of PD, CCL18 has been shown to be increased and is believed to be important in the modulation of the inflammatory response and tissue repair.^21^ A previous study demonstrated that CCL18 was decreased in PD progression.^22^ However, there is limited understanding of the precise mechanisms through which CCL18 impacts the osteogenic differentiation of PDLSCs.

Therefore, the present study aimed to investigate the role of M2 macrophage-derived exosomes in regulating the osteogenic differentiation of human PDLSCs, with a focus on the involvement of CCL18. The authors hypothesized that M2-exos promote the osteogenic differentiation of PDLSCs via the secretion of CCL18. The present findings provide new insights into the mechanisms underlying M2-exo-mediated periodontal regeneration and highlight the potential of M2-exos as a novel therapeutic tool for periodontitis.

## Materials and methods

### THP-1 cell culture and polarization induction

In this study, THP-1 human monocytes and hPDLSCs were utilized. THP-1 macrophages, obtained from ATCC (Manassas, VA, USA), were cultured in DMEM (Grand Island, NY, USA) supplemented with 10 % Fetal Bovine Serum (FBS, Gibco) and 1 % penicillin/streptomycin (Gibco). The cells were incubated at 37 °C in a humidified environment with 5 % CO_2_. Accordingly, to previous study,^23^ THP-1 cells were treated with 20 ng/mL IL-4 and 20 ng/mL of IL-13 for 12 h to induce M2 polarization. To induce M1 polarization of THP-1, the cells were treated with 200 ng/mL LPS for 12 h. hPDLSCs were provided with Procell (Wuhan, China). M0 THP-1 cells were cultured in normal DMEM. Studies followed the STROBE Statement.

### Isolation of exosomes

According to a previously established protocol, exosomes derived from M2-polarized THP-1 macrophages (M2-exo) were isolated through ultracentrifugation.^24^ The medium supernatant was centrifuged at 300 × g for 15 min, and then centrifuged at 3000 × g for 15 min, and followed by 20,000 × g for 70 min. Subsequently, the exosomes were purified by centrifugation at 120,000 × g for 70 min. The purified exosomes were preserved at −80° for further study.

### Identification of M2-exo

M2-exosomes were characterized using both Transmission Electron Microscopy (TEM) and Nanoparticle Tracking Analysis (NTA). For TEM analysis, exosome samples were applied onto a copper mesh for 3 min, followed by staining with 2 % (w/v) phosphotungstic acid for 3 min, and subsequently examined using a JEOL TEM system (Tokyo, Japan). The size distribution of exosomes was determined through NTA measurements.

Western blot analysis was performed to evaluate the levels of exosome surface markers CD63 and CD9. Protein samples were extracted from both M2 macrophages and exosomes, followed by quantification using a BCA kit (Thermo Fisher, Waltham, MA, USA). After separation via SDS-PAGE, the proteins were transferred to membranes and incubated with specific primary antibodies targeting CD63 or CD9. Afterwards, the proteins were treated with corresponding secondary antibodies. Protein bands were visualized using the ECL kit (Thermo Fisher).

### HPDLSCs cell culture and treatment

To examine how THP-1 macrophage polarization influences the osteogenic differentiation capacity of hPDLSCs, the hPDLSCswere co-culture with M0, M1 and M2 polarization THP-1 in DMEM (Grand Island) containing 10 % FBS (Gibco) and 1 % penicillin/streptomycin (Gibco) and incubated at 37° and 5 % CO_2_, respectively. For M2-exo treatment, 10 μg/mL of exosomes were added to the DMEM, and hPDLSCs were cultured for 24 h. For the induction of osteogenic differentiation in hPDLSCs, cells were cultured in the medium containing 50 μg/mL β-ascorbic acid, 20 nM dexamethasone, and 8 mM β-glycerol phosphate for subsequent experimental procedures.

For the knockdown of CCL8, short hairpin RNA specifically targeting CCL8 (sh-CCL8) along with its corresponding Negative Control (sh-NC) were obtained from GenePharma (Shanghai, China). Human Periodontal Ligament Stem Cells (hPDLSCs) were plated in 24-well plates (4 × 10^4^ cells/well). Transfection was conducted using Lipofectamine 2000 (Invitrogen, Carlsbad, CA, USA) following the manufacturer's instructions. The transfected cells were collected 48 h post-transfection for further analysis.

### Reverse transcription quantitative real-time PCR (RT-qPCR)

Total RNA was isolated from PDLSCs and THP-1 cells using TRIzol reagent (Invitrogen, Carlsbad, USA). Following RNA quantification, cDNA synthesis was performed using a cDNA synthesis kit (Vazyme, Nanjing, China). Quantitative Real-Time PCR (RT-PCR) was subsequently conducted with the HiScript® Q RT SuperMix for qPCR kit (Vazyme), using GAPDH as the internal reference gene. The expression of mRNA was calculated using the 2^-ΔΔCt^ method.^25^ All experimental procedures were performed in triplicate to ensure reproducibility.

### Alkaline phosphatase (ALP) staining and ALP activity

According to a previous study,^26^ following 7-days of osteogenic differentiation induction, Alkaline Phosphatase (ALP) staining was conducted using a commercial ALP staining kit (Beyotime, Shanghai, China). According to the instruction, the hPDLSCs were incubated with staining solution for 30 min at room temperature under light-protected conditions. Subsequently, ALP activity was quantitatively assessed using an ALP assay kit (Beyotime), with absorbance measurements taken at 405 nm.

### Alizarin red S (ARS) staining

According to a previous study,^27^ following 14 or 21-days of osteogenic differentiation induction, mineralized nodule formation was assessed using Alizarin Red S (ARS) staining with a commercial kit (Beyotime). The hPDLSCs were first rinsed with PBS and subsequently fixed for 20 min. The cells were then incubated with ARS staining solution at room temperature for 30 min, followed by microscopic examination. For quantitative analysis, the ARS stain was solubilized using cetylpyridinium chloride, and the absorbance was detected spectrophotometrically at 562 nm.

### Statistical analysis

All experiments were repeated a minimum of three times independently. Data were analyzed using GraphPad Prism 8 and presented as mean ± SD. Multiple group comparisons were performed using one-way ANOVA, with *p* < 0.05 considered statistically significant.

## Results

### M2 macrophages promoted osteogenic differentiation of hPDLSCs

**Fig. 1:**
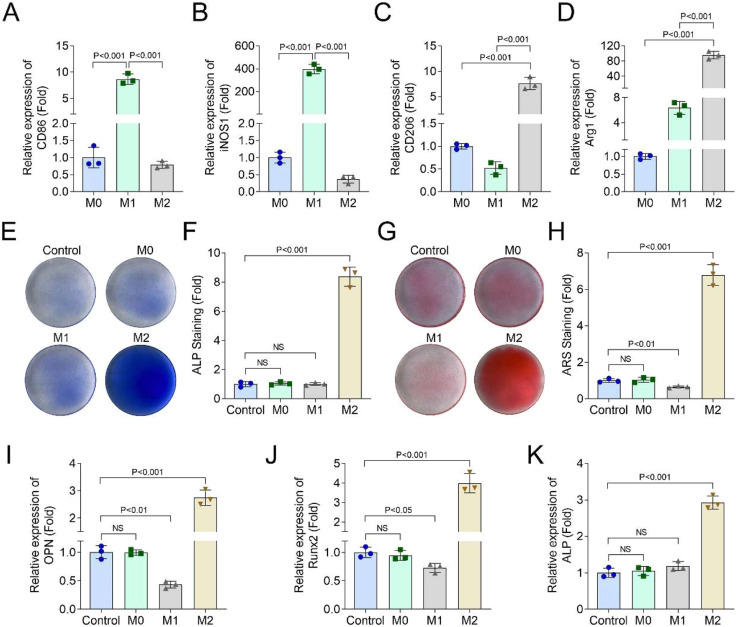
M2 macrophages promoted osteogenic differentiation of hPDLSCs. THP-1 cells were treated with 20 ng/mL IL-4 and 20 ng/mL IL-13 for M2 polarization, and treated with LPS for M1 polarization. The mRNA levels of CD86 (A), iNOS1 (B), CD209 (C) and Arg1 (D) in M0, M1, and M2 THP-1 cells were detected by RT-qPCR assay. hPDLSCs were co-cultured with M1 and M2 THP-1 cells. ALP (E and F) and ARS (G and H) staining was performed to analyze the osteogenic differentiation of hPDLSCs. The mRNA levels of OPN (I), Runx2 (J) and ALP (K) in hPDLSCs were detected by RT-qPCR assay (*n* = 3).

Firstly, after M1 and M2 polarization induction, the authors found that the CD86 and iNOS1 mRNA levels were significantly increased in M1 THP-1 cells, and decreased in M2 THP-1 cells (Fig. 1A and B); the CD209 and Arg1 mRNA levels were significantly decreased in M1 THP-1 cells, and increased in M2 THP-1 cells (Fig. 1C and D). ALP (Fig. 1E and F) and ARS (Fig. 1G and H) staining showed that M1 THP-1 cells suppressed the osteogenic differentiation of hPDLSCs and M2 THP-1 cells promoted hPDLSCs' osteogenic differentiation. Additionally, after 21-days of osteogenic differentiation induction, ARS staining also found that M2 macrophages could promote the osteogenic differentiation of hPDLSCs (Supplementary Fig. 1 A). Besides, the authors also found that M2 THP-1 cells increased the OPN, Runx2, and ALP levels in hPDLSCs, while M1 THP-1 cells decreased the OPN and Runx2 levels (Fig. 1I‒K). These results indicated that M2 THP-1 cells promoted osteogenic differentiation of hPDLSCs.

### Identification of M2-exo

**Fig. 2:**
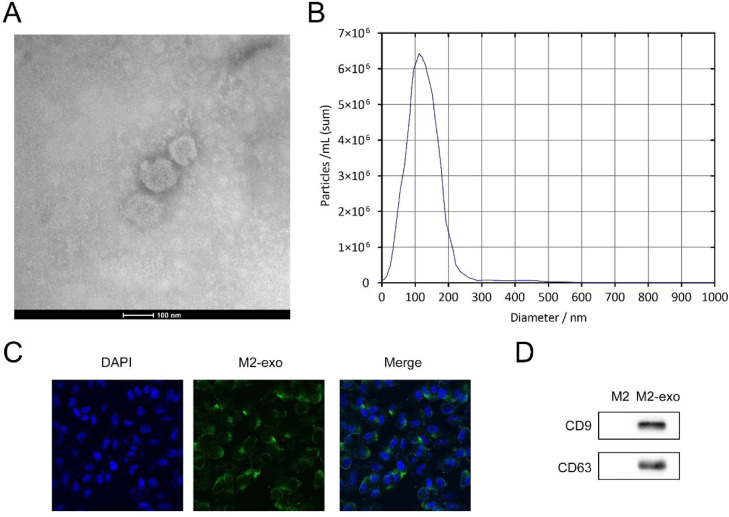
Identification of M2-exo. TEM (A) and NTA analysis (B) results of M2-exo. (C) PKH67 staining of M2-exos in hPDLSCs. (D) The protein levels of CD9 and CD63 in M2-exos were detected by western blot (*n* = 3).

As depicted in Fig. 2A, TEM analysis indicated that M2-exos exhibit a spherical morphology characterized by a double membrane structure. NTA measurements further demonstrated that the particle size distribution of M2-exos predominantly ranged between 100‒200 nm (Fig. 2B). Cellular uptake experiments using PKH67 staining confirmed the internalization of M2-exos by hPDLSCs (Fig. 2C). Additionally, western blot analysis of exosomal marker proteins revealed significant upregulation of CD9 and CD63 expression in M2-exos (Fig. 2D). Collectively, these findings provide substantial evidence that the isolated particles from M2 macrophages are indeed exosomes.

### M2-exo promoted the osteogenic differentiation of hPDLSCs

**Fig. 3:**
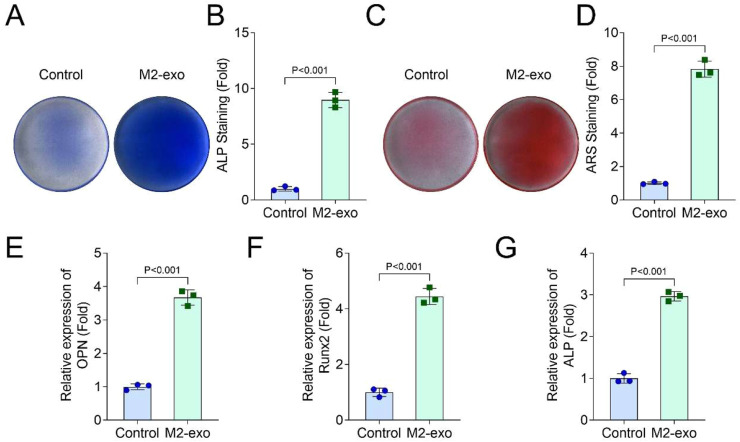
M2-exo promoted the osteogenic differentiation of hPDLSCs. The hPDLSCs were treated with 10 μg/mL M2-exos. ALP (A and B) and ARS (C and D) staining was performed to analyze the osteogenic differentiation of hPDLSCs. The mRNA levels of OPN (E), Runx2 (F) and ALP (G) in hPDLSCs were detected by RT-qPCR assay (*n* = 3).

Then, the M2-exos were administered to hPDLSCs. ALP (Fig. 3A and B) and ARS (Fig. 3C and D) staining suggested that M2-exos facilitated hPDLSCs osteogenic differentiation. Additionally, after 21-days of osteogenic differentiation induction, ARS staining also found that M2-exos could promote the osteogenic differentiation of hPDLSCs (Supplementary Fig. 1 B). The PCR results demonstrated that M2-exos elevated the OPN (Fig. 3E), Runx2 (Fig. 3F) and ALP (Fig. 3G) levels in hPDLSCs.

### CCL18 knockdown reversed the role of M2-exo in hPDLSCs

**Fig. 4:**
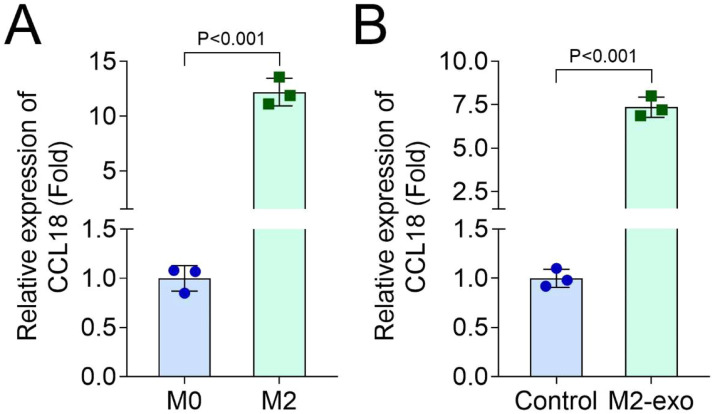
CCL18 was increased in M2-exo and M2-exo treated hPDLSCs. The mRNA levels of CCL18 in M2-exo (A) and M2-exo treated hPDLSCs (B) were detected by RT-qPCR (*n* = 3).

**Fig. 5:**
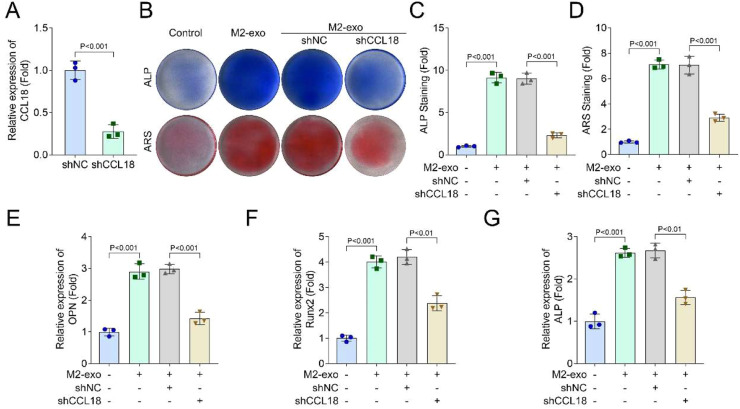
CCL18 knockdown reversed the role of M2-exo in hPDLSCs. The hPDLSCs were treated with 10 μg/mL M2-exos and transfected with shCCL18. The knockout efficiency of shCCL18 was detected by RT-qPCR assay (A). (B‒D) ALP and ARS staining were performed to analyze the osteogenic differentiation of hPDLSCs. The mRNA levels of OPN (E), Runx2 (F), and ALP (G) in hPDLSCs were detected by RT-qPCR assay (*n* = 3).

Subsequently, the authors confirmed that CCL18 was upregulated in M2 THP-1 cells (Fig. 4A) and M2-exos treated hPDLSCs (Fig. 4B). Then, in M2-exos treated hPDLSCs, sh-CCL18 was transfected to knock down the CCL18 levels (Fig. 5A). Then, after CCL18 knockdown, ALP and ARS staining showed that CCL18 knockdown suppressed the osteogenic differentiation of M2-exos treated hPDLSCs (Fig. 5B‒D). Additionally, after 21-days of osteogenic differentiation induction, ARS staining also found that CCL18 knockdown could inhibit the osteogenic differentiation of M2-exos treated hPDLSCs (Supplementary Fig. 1C). In addition, the PCR results showed that CCL18 knockdown decreased the OPN (Fig. 5E), Runx2 (Fig. 5F) and ALP (Fig. 5G) levels in M2-exos treated hPDLSCs.

## Discussion

PD is defined as a progressive inflammatory condition leading to the gradual deterioration of tooth-supporting tissues.^15^ Exosomes are small extracellular vesicles that are actively secreted by a diverse range of cell types, including M2 macrophages, and have emerged as key mediators of intercellular communication and tissue regeneration.^21^^,^^28^ This study sought to examine the regulatory influence of M2-exos on the osteogenic differentiation potential of hPDLSCs and their capacity to attenuate the advancement of periodontal pathology. These findings revealed that M2-exos markedly promoted the osteogenic differentiation capacity of hPDLSCs, as indicated by the elevated expression of key osteogenic markers, including Runx2, OPN, and ALP. As reported by previous studies, the effects of M2-exos on periodontitis therapy were achieved through the secretion of multiple substances. For example, Cui et al.^8^ revealed that melatonin secreted from M2-exos restored the osteogenic and cementogenic differentiation potential of inflammatory hPDLSCs through the suppression of excessive endoplasmic reticulum stress, suggesting that exosomes derived from M2 macrophages hold significant promise for the regeneration of inflammatory periodontal tissues. In addition, Chen et al.^13^ found that M2 macrophages promoted osteogenesis and inhibited osteoclastogenesis in vitro and in vivo. M2-Exos increased the level of IL-10 in bone marrow stromal cells by directly delivering exosomal IL-10 mRNA to target cells. Based on these studies, the authors propose a hypothesis that in the present study, the promotion of M2-Exos on cell osteogenic differentiation is achieved through the secretion of what substances?

CCL18, a chemokine primarily produced by alternatively activated M2 macrophages, has gained attention due to its potential regulatory role in inflammation and tissue repair.^29^^,^^30^ Previous studies revealed that macrophage-derived CCL18 participated in various niological behavior of cells. In breast cancer, Chen et al.^31^ found that tumor-associated macrophages could affect cancer progression and metastasis by producing CCL18, which enhances the invasiveness of cancer cells through the induction of integrin clustering and the subsequent reinforcement of their adhesion to the extracellular matrix. The same role of macrophage-derived CCL18 also confirmed in osteosarcoma,^32^ glioma,^33^ and lung cancer.^34^ Besides tumors, in microglia, CCL18 was demonstrated to exhibit anti-inflammatory activity, promote the phagocytic function of microglia, which further participated in neural development, homeostasis, and repair mechanisms.^35^ Interestingly, recent research has found a close relationship between hPDSCLs and macrophage M2 polarization. The co-culture of hPDSCLs and Macrophages induced the production of IL-10, TGF-β, and CCL18.^22^ Therefore, the authors speculated whether the production of CCL18 is due to M2-Exos. Here, we found that CCL18 was increased in M2 THP-1 cells and M2-exos treated hPDLSCs. Knockdown of CCL18 reversed the effects of M2-exos on osteogenic differentiation, and Runx2, OPN, and ALP expression levels of hPDLSCs. These results indicated that the promotion of M2 exos on osteogenic differentiation of hPDLSCs is caused by the secretion of CCL18.

Nevertheless, this study is not without limitations. The current findings are primarily based on in vitro evidence, and further validation in animal models of periodontitis is essential to confirm the therapeutic potential of M2-exos. Looking forward, this study paves the way for several promising research directions. First, given the complex cargo of exosomes, it is plausible that other signaling molecules within M2-exos, besides CCL18, may also contribute to the osteogenic differentiation of PDLSCs. Future proteomic or transcriptomic analyses of M2-exos could identify additional candidates. Second, the downstream signaling pathways activated by CCL18 in PDLSCs remain to be fully elucidated. Investigating receptors and intracellular cascades, such as the PI3K/Akt or MAPK/ERK pathways, will provide deeper mechanistic insights. Finally, translating these findings into therapeutic applications, for instance by developing engineered exosomes enriched with pro-osteogenic factors or by designing agonists that enhance the secretion of beneficial molecules like CCL18 from M2 macrophages, represents a compelling frontier for future research. These directions will collectively lay a solid foundation for harnessing macrophage-derived exosomes as a novel regenerative therapy for periodontal disease.

Collectively, the authors revealed that M2 macrophages enhanced the osteogenic differentiation of hPDLSCs. Furthermore, M2 macrophage-derived exosomes were found to play an essential role in the osteogenic differentiation of hPDLSCs. This study demonstrated that M2-exos secrete CCL18, which significantly upregulates CCL18 expression in PDLSCs. The enhanced osteogenic differentiation, demonstrated by the elevated expression of osteogenic markers including Runx2, OCN, and ALP, was correlated with the upregulation of CCL18.

## Ethics approval and consent to participate

Not applicable.

## Consent for publication

Not applicable.

## Authors' contributions

All authors participated in the design, interpretation of the studies and analysis of the data and review of the manuscript. Z F drafted the work and revised it critically for important intellectual content; Y Z and J Z were responsible for the acquisition, analysis and interpretation of data for the work; Z Y and Z F made substantial contributions to the conception or design of the work. All authors read and approved the final manuscript.

## Funding

The authors declare that no funds, grants, or other support were received during the preparation of this manuscript.

## Declaration of competing interest

The authors declare no conflicts of interest.

## Data Availability

The datasets used and/or analyzed during the current study are available from the corresponding author on reasonable request.

## Appendix Supplementary materials

Image, application 1
