# Supplementary material for: M2 Macrophages derived exosomes promoted Periodontal ligament stem cells osteogenic differentiation through secreting CCL18
Source: Clinics (Sao Paulo). 2026 Mar 27;81:100916. doi: 10.1016/j.clinsp.2026.100916 (PMC13054062; doi:10.1016/j.clinsp.2026.100916)

**CLINICS-D-25-00742_ Supplementary Material**

**Supplementary Figure 1 ARS staining of hPDLSCs after 21-days of osteogenic differentiation induction.** (A) hPDLSCs were co-cultured with M1 and M2 THP-1 cells, ARS staining was performed after 21-days. (B) hPDLSCs were treated with 10 μg/mL M2-exos, ARS staining was performed after 21-days. (C) The hPDLSCs were treated with 10 μg/mL M2-exos and transfected with shCCL18; ARS staining was performed after 21 days.


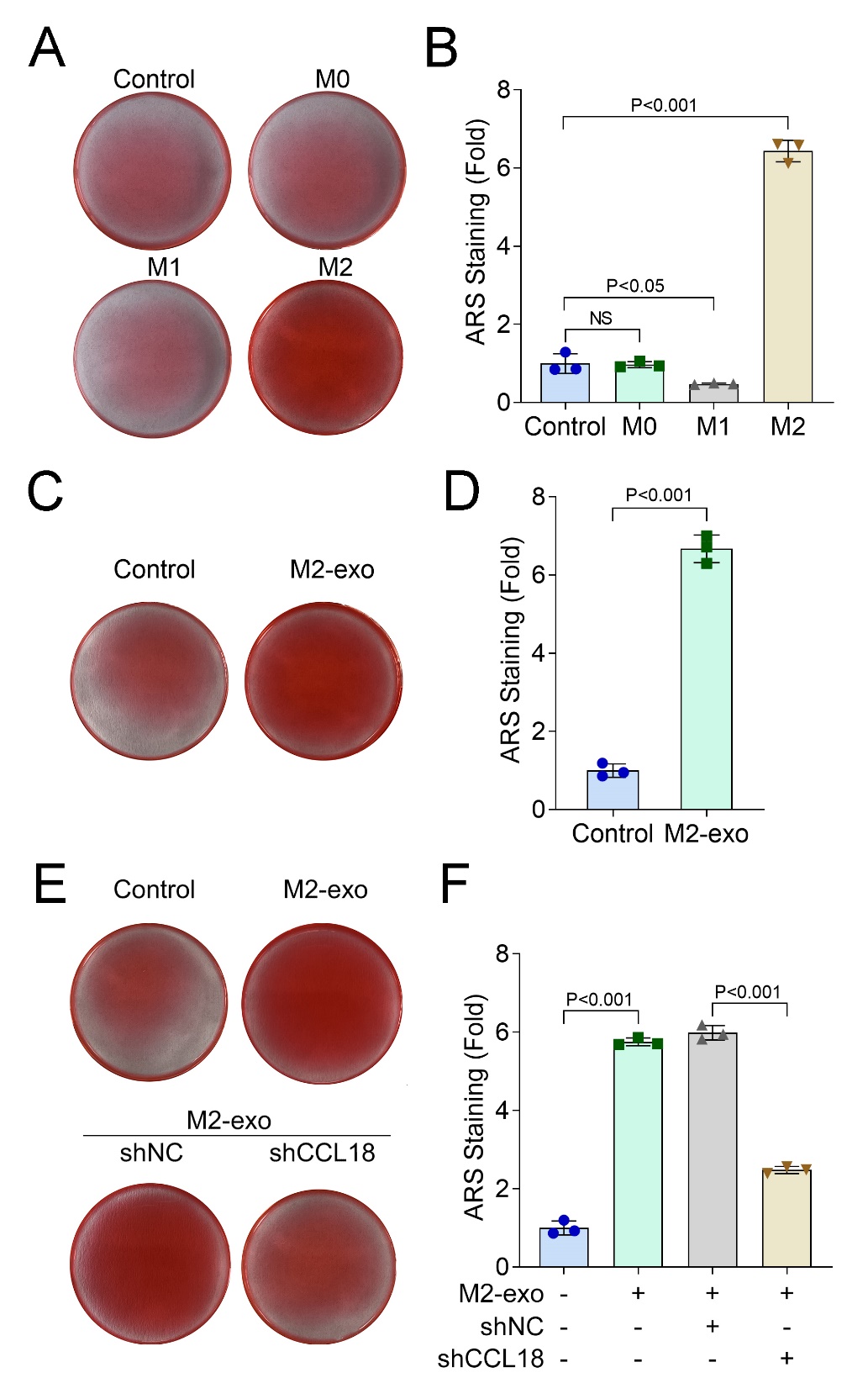

Supplement: Supplementary file 1 [file mmc1.docx]
